# Supplementary material for: Enhanced Arbovirus Surveillance with High-Throughput Metatranscriptomic Processing of Field-Collected Mosquitoes
Source: Viruses. 2022 Dec 11;14(12):2759. doi: 10.3390/v14122759 (PMC9782886; doi:10.3390/v14122759)
Supplement: Supplementary file 1 [file viruses-14-02759-s001.zip › viruses-1964990-supplementary.pdf]

Table S1. Mosquito counts for the 21 traps used in this study, sampled over seven weeks (45 – 51) in 2016 in Gannawarra, Mildura and Wellington. The traps were first subsampled for cell culture-based arbovirus screening by the Victorian Arbovirus Disease Control Program (VADCP), and then the rest of the trap was split into differently sized subsamples for metatranscriptomic sequencing. The sequencing subsample sizes consisted of 100, 1000 and the remaining mosquitoes in each trap. The mosquito counts are based on weight.

**Location**	**Week**	**Estimated total mosquito count**	**Mosquitoes screened via cell culture**	**Mosquitoes screened via sequencing**	**Number of sequencing subsamples**
Gannawarra	45	4595	370	4225	6
	46	2502	152	2350	4
	47	1992	148	1844	3
	48	1652	182	1470	3
	49	1157	351	806	2
	50	2154	347	1807	3
	51	3814	358	3456	5
Mildura	45	618	340	278	2
	46	2014	149	1865	3
	47	2051	152	1899	3
	48	3958	349	3609	5
	49	2356	350	2006	3
	50	1189	348	841	2
	51	1706	350	1356	3
Wellington	45	4829	349	4480	6
	46	1535	152	1383	3
	47	13,659	264	13,395	15
	48	1497	222	1275	3
	49	5226	336	4890	6
	50	1186	366	820	2
	51	2528	350	2178	4
**Total:**		**62,218**	**5985**	**56,233**	**86**

Table S2: The number of 3 mm glass beads (Sigma-Aldrich) and volume of Buffer AVL (Qiagen) that was added to each sample based on the number of mosquitoes present.

**Mosquito no.**	**Bead no.**	**Buffer AVL (mL)**
1-100	10	1
101-200	13	2
201-300	16	3
301-400	19	4
401-500	22	5
501-600	25	6
601-700	28	7
701-800	31	8
801-900	34	9
901-1000	37	10

Table S3: Details of the reverse transcription quantitative PCR (RT-qPCR) assays used to screen trap subsamples for the following arboviruses: Ross River virus (RRV), Trubanaman virus (TRUV), Umatilla virus (UMAV), Sindbis virus (SINV) and Wongorr virus (WGRV).

Virus	Primer/probe name	Primer/probe sequence (5' → 3')	Primer conc. (µM)	Target	Amplicon size (bp)	Reference	PCR kit	PCR cycling conditions	RNA (µL)	Additional reagents
RRV	RRVE2F	ACGGAAGAAGGGATTGAGTACCA	7.5	E2	67	[45]	AgPath-ID One-Step RT-PCR Kit (Applied Biosystems)	30' 48°C; 10' 95°C; 40 x (15" 95°C, 1' 60°C)	5	N/A
	RRVE2R	TCGTCAGTTGCGCCCATA	7.5							
	RRVE2Prob	CAACAACCCGCCGGTCCGC	5							
TRUV	MUR-BUC-TRUV S -F	TGGAGACCTGGAAGTTGTTATTCA	10	N	487	[28]		30' 48°C; 10' 95°C; 40 x (15" 95°C, 30" 56°C, 45" 60°C); 15" 95°C; 1' 56°C; 15" 95°C		10X SYBR Green I (Invitrogen)
	MUR-BUC-TRUV S 718 -R	ATTGTCTTGCAGCTTCTGACATG								
UMAV	SLOV S2 602F	TGAACCGGCCGATACAGAAT		T2	251	[47]		30' 48°C; 10' 95°C; 40 x (15" 95°C, 1' 60°C); 15" 95°C; 1' 60°C; 15" 95°C		
	SLOV S2 852R	TGAGGGATTTGGTGGTAATGTG								
SINV	VIR966-F	TCCATGCTAATGCTAGAGCGTTTTCGCA		nsP1	98	[46]	Power SYBR Green RNA-to-Ct 1-Step Kit (Applied Biosystems)	30' 48°C; 10' 95°C; 40 x (15" 95°C, 1' 60°C); 15" 95°C; 15" 60°C; 15" 95°C	2	N/A
	VIR966-R	TGGCGCACTTCCAATGTCCAGGAT								
WGRV	FC54	TAATGGGTGGCAGTGCG		VP3	214	In-house	SensiFAST SYBR Lo-ROX One-Step Kit (Bioline)	15' 45°C; 2' 95°C; 40 x (5" 95°C, 10" 58°C); 10" 72°C; 15" 95°C; 1' 58°C; 15" 95°C	4	
	RC2 53	GCTGTGACATGAGGTTCATGTAATT								

Table S4: Arbovirus detections made by metatranscriptomic sequencing in the 21 mosquito traps from Gannawarra, Mildura and Wellington over seven weeks (45 – 51) in 2016 using three different positive detection criteria: (**A**) Fold Coverage by Reads ratio (FCR-r), resulting in 9 detections; (**B**) Percent Coverage by Contigs ratio (%CC-r), resulting in 15 detections; (**C**) Percent Coverage by Reads ratio (%CR-r), resulting in 22 detections. Ratios ≥ 2 were considered a positive detection.

**(A)**	**FCR-r**	**Gannawarra**							**Mildura**							**Wellington**						
		**45**	**46**	**47**	**48**	**49**	**50**	**51**	**45**	**46**	**47**	**48**	**49**	**50**	**51**	**45**	**46**	**47**	**48**	**49**	**50**	**51**
	Ross River virus					■						■						■				
	Sindbis virus							■							■							
	Trubanaman virus		■								■				■							
	Umatilla virus													■								
	Wongorr virus																					

**(B)**	**%CC-r**	**Gannawarra**							**Mildura**							**Wellington**						
		**45**	**46**	**47**	**48**	**49**	**50**	**51**	**45**	**46**	**47**	**48**	**49**	**50**	**51**	**45**	**46**	**47**	**48**	**49**	**50**	**51**
	Ross River virus					■	■					■						■				■
	Sindbis virus						■	■							■							
	Trubanaman virus		■	■							■	■			■							
	Umatilla virus													■	■							
	Wongorr virus																					

**(C)**	**%CR-r**	**Gannawarra**							**Mildura**							**Wellington**						
		**45**	**46**	**47**	**48**	**49**	**50**	**51**	**45**	**46**	**47**	**48**	**49**	**50**	**51**	**45**	**46**	**47**	**48**	**49**	**50**	**51**
	Ross River virus					■	■					■						■		■		■
	Sindbis virus						■	■							■							
	Trubanaman virus		■	■						■	■	■			■							
	Umatilla virus											■		■	■							
	Wongorr virus							■				■		■	■							

Table S5: Arbovirus detections in mosquito subsamples based on reverse transcription quantitative PCR compared to metatranscriptomic sequencing (MSeq). Only Ct values are reported for the Ross River virus (RRV) assay as it is probe-based, whereas both Ct and Tm values are reported for the Sindbis virus (SINV), Trubanaman virus (TRUV), Umatilla virus (UMAV), and Wongorr virus (WGRV) assays as they are SYBR-based assays. The MSeq Percent Coverage by Reads (%CR) is provided for each detection.

Virus	Location	Week	Subsample	Mosq. no.	Ct	Tm	MSeq positive	%CR
RRV	Gannawarra	49	1	100	39.99	-	No	0.00
RRV	Gannawarra	49	R	706	27.97	-	Yes	99.43
RRV	Gannawarra	50	2	1000	32.56	-	Yes	29.61
RRV	Gannawarra	50	R	707	37.74	-	No	0.00
RRV	Mildura	48	2	1000	39.82	-	No	1.73
RRV	Mildura	48	4	1000	27.8	-	Yes	98.22
RRV	Mildura	49	2	1000	39.87	-	No	0.00
RRV	Wellington	47	2	1000	28.87	-	Yes	98.28
RRV	Wellington	47	6	1000	38.56	-	Yes	4.32
RRV	Wellington	47	11	1000	39.39	-	Yes	2.15
RRV	Wellington	49	2	1000	35.83	-	Yes	3.83
RRV	Wellington	51	2	1000	34.3	-	Yes	45.22
SINV	Gannawarra	50	2	1000	*Negative*		Yes	10.06
SINV	Gannawarra	50	R	707	39.84	82	Yes	9.17
SINV	Gannawarra	51	2	1000	38.78	82	Yes	98.86
SINV	Gannawarra	51	3	1000	39.21	82	Yes	98.30
SINV	Gannawarra	51	4	1000	*Negative*		Yes	9.20
SINV	Mildura	51	2	1000	39.91	82	Yes	98.83
TRUV	Gannawarra	46	1	100	33.66	83	No	1.53
TRUV	Gannawarra	46	2	1000	27.49	83.5	Yes	28.73
TRUV	Gannawarra	46	3	1000	23.56	83	Yes	86.02
TRUV	Gannawarra	46	R	250	28.92	83	Yes	3.77
TRUV	Gannawarra	47	R	744	26.52	83.5	Yes	49.92
TRUV	Gannawarra	51	2	1000	31.2	83	No	0.00
TRUV	Mildura	46	2	1000	30.78	83	No	0.00
TRUV	Mildura	46	R	765	28.89	83.5	Yes	2.35
TRUV	Mildura	47	1	100	27.69	83	Yes	3.42
TRUV	Mildura	47	2	1000	24.58	83.5	Yes	32.12
TRUV	Mildura	47	R	799	19.07	83.5	Yes	98.43
TRUV	Mildura	48	2	1000	26.68	83.5	Yes	3.99
TRUV	Mildura	48	R	509	25.11	83.5	Yes	34.60
TRUV	Mildura	49	2	1000	30.29	83	No	1.52
TRUV	Mildura	51	1	100	26.79	83.5	Yes	14.87
TRUV	Mildura	51	2	1000	20.82	83.5	Yes	93.36
TRUV	Mildura	51	R	256	29.96	83	No	0.00
UMAV	Mildura	48	1	100	33.55	82	No	0.00
UMAV	Mildura	48	2	1000	31.03	82	Yes	10.72

UMAV	Mildura	50	R	741	26.04	81.5	Yes	55.89
UMAV	Mildura	51	2	1000	32.78	82	Yes	28.51
WGRV	Gannawarra	51	2	1000	34.7	86.5	Yes	30.34
WGRV	Mildura	48	2	1000	33.47	86.5	Yes	17.11
WGRV	Mildura	49	1	100	33.36	86	No	0.00
WGRV	Mildura	49	2	1000	33.98	86	No	0.00
WGRV	Mildura	50	R	741	33.73	85.5	Yes	7.75
WGRV	Mildura	51	2	1000	33.72	87	Yes	9.58

R = remainder subsample

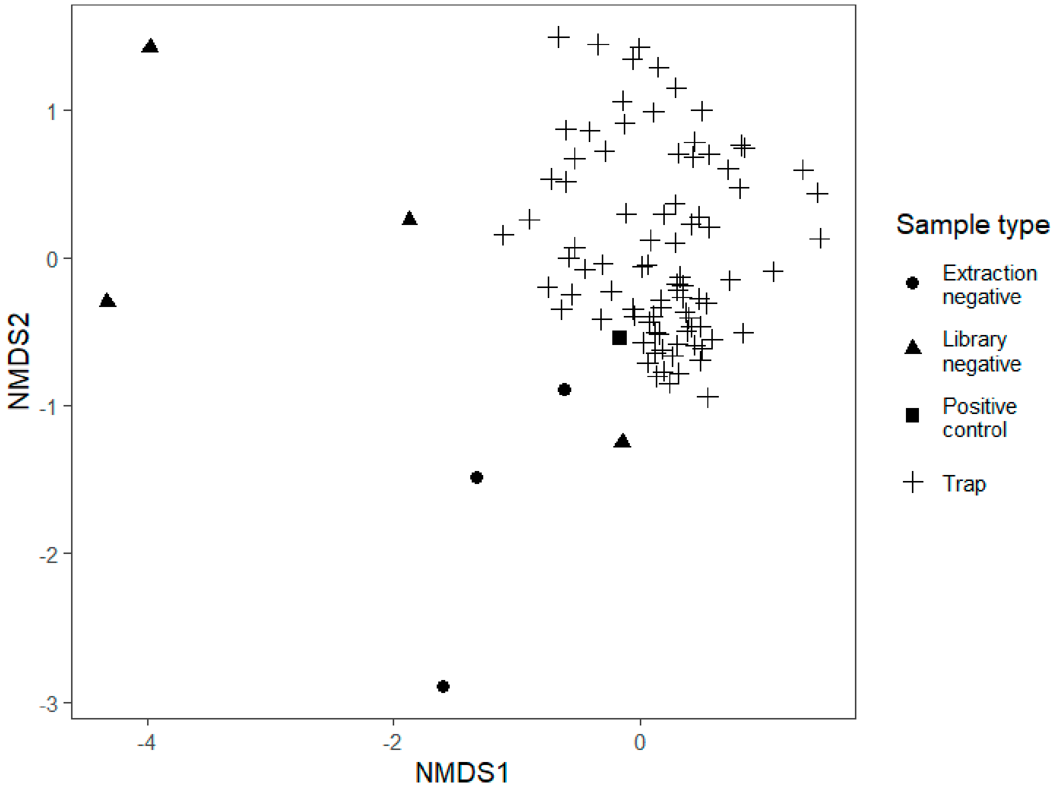

Figure S1: Non-metric multidimensional scaling (NMDS) based on Bray-Curtis dissimilarity of the contigs in trap samples (crosses), the positive control sample (square), library negative control samples (triangles), and extraction negative control samples (circles).

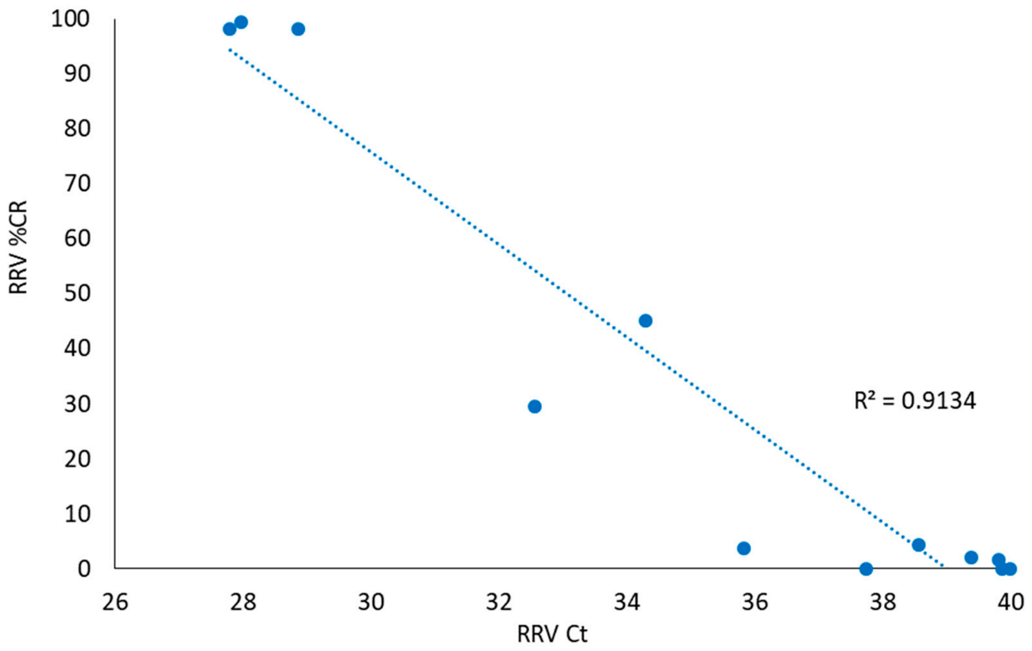

Figure S2: The relationship between Ross River virus (RRV) Ct values produced by reverse transcription quantitative PCR and RRV Percent Coverage by Reads (%CR) produced by metatranscriptomic sequencing.

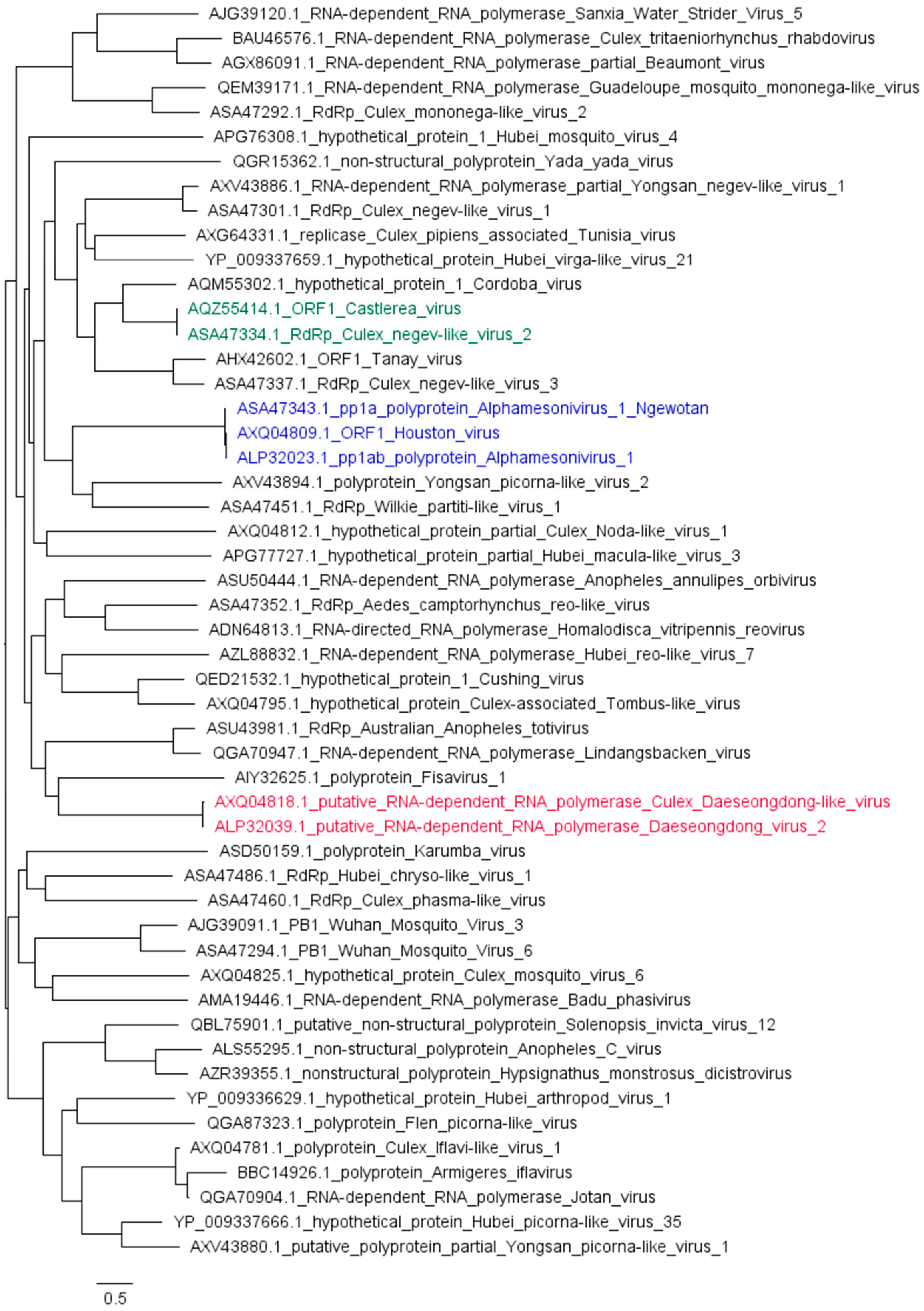

Figure S3: A maximum likelihood tree of reference GenBank RNA-dependent RNA polymerase (RdRp) sequences of the 51 viruses that were a BLASTx match for trap contigs as part of the broader virome analysis. Highly similar or identical reference RdRp sequences have been highlighted in distinct colours and indicate possible redundancies in virus detection.

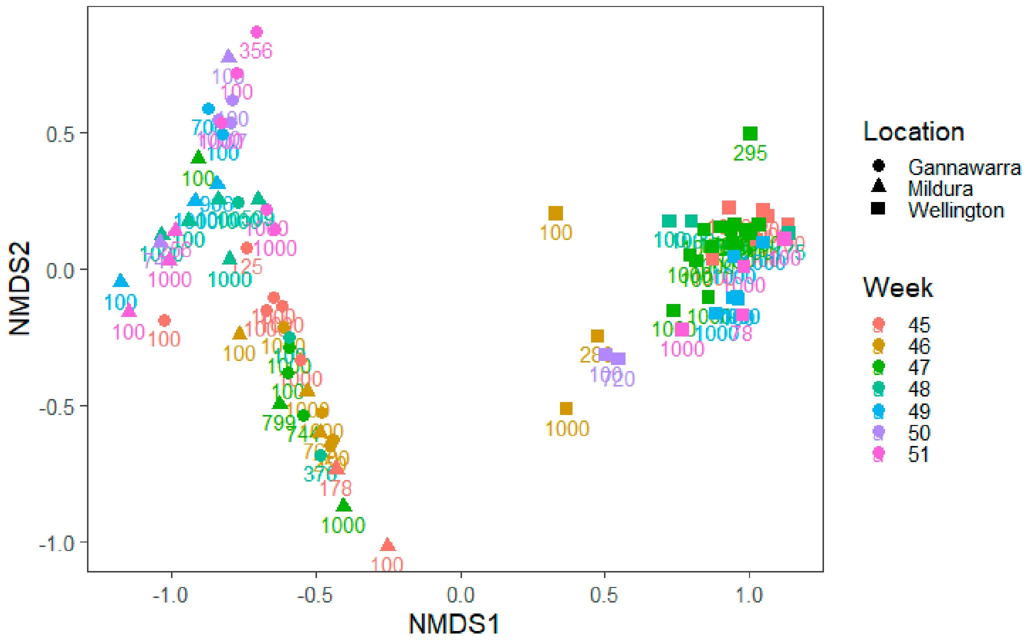

(A)

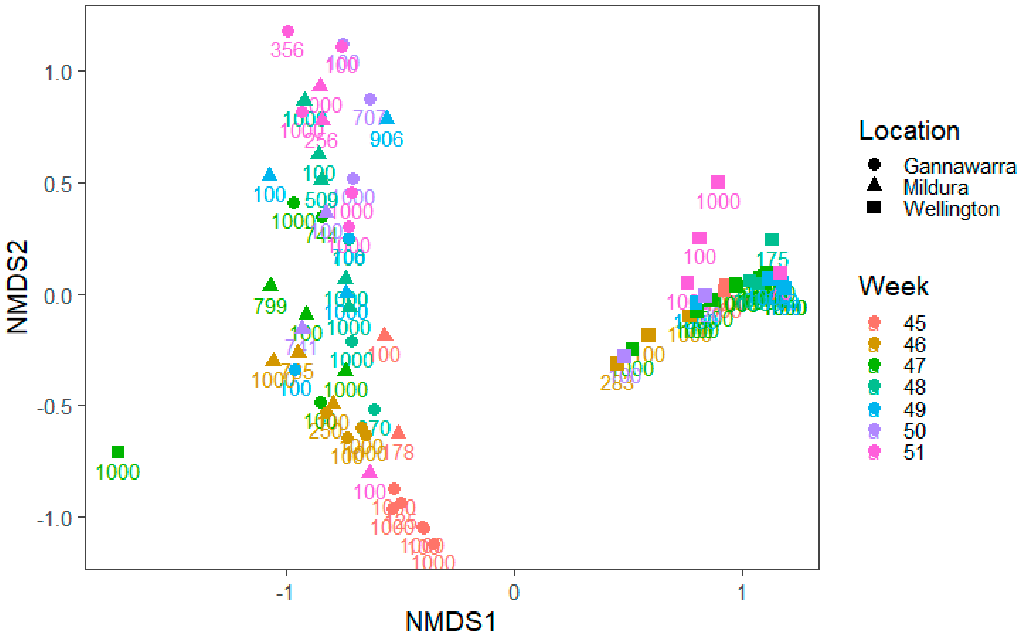

(B)

Figure S4: Non-metric multidimensional scaling (NMDS) based on Bray-Curtis dissimilarity of the (**A**) viruses and (**B**) mosquitoes in the trap subsamples from three locations over seven weeks showing a separation between coastal (Wellington) and inland (Gannawarra and Mildura) samples. The number of mosquitoes in each subsample is also shown.
